# Pulmonary Artery and Vein Morphology as an Imaging Biomarker for the Diagnosis of Pulmonary Hypertension

**DOI:** 10.3390/diagnostics16040619

**Published:** 2026-02-20

**Authors:** Nedim Christoph Beste, Alexander Christian Bunck, Jonathan Kottlors, Robert Peter Wawer Matos Reimer, Jan Robert Kröger, Thomas Schömig, Lenhard Pennig, Kenan Kaya, Carsten Gietzen, Nils Große-Hokamp, Martin Urschler, Horst Olschewski, Stephan Rosenkranz, Florian J. Fintelmann, Michael Pienn, Roman Johannes Gertz

**Affiliations:** 1 Department of Radiology, Faculty of Medicine, University Hospital Cologne, University of Cologne, 50937 Cologne, Germany; 2 Department of Radiology, Neuroradiology and Nuclear Medicine, Johannes Wesling University Hospital, Ruhr University Bochum, 44801 Bochum, Germany; 3 Institute for Medical Informatics Statistics and Documentation, Medical University of Graz, 8010 Graz, Austria; 4 Faculty of Medicine, Sigmund Freud Private University, 1020 Vienna, Austria; 5 Department Pneumology, Intensive Care Medicine and Sleep Medicine, Charité University Medicine, 10117 Berlin, Germany; 6 ERN Lung Pulmonary Hypertension National Expert Center, 8010 Graz, Austria; 7 Department of Cardiology, Heart Center, Faculty of Medicine, University of Cologne, 50937 Cologne, Germany; 8 Thoracic Imaging & Intervention, Massachusetts General Hospital, Boston, MA 02114, USA; 9 Division of Pulmonology, Department of Internal Medicine, Lung Research Cluster, Medical University of Graz, 8010 Graz, Austria; 10 Ludwig Boltzmann Institute for Lung Vascular Research, 8010 Graz, Austria

**Keywords:** pulmonary hypertension, pulmonary vascular morphology, segmentation knowledge-based algorithms, computed tomography pulmonary angiography

## Abstract

**Background/Objectives**: To evaluate whether peripheral pulmonary artery and vein morphology improves image-based diagnosis of pulmonary hypertension (PH), in accordance with the recently updated hemodynamic definition. **Methods**: 229 patients underwent CT pulmonary angiography (CTPA) within 30 days of RHC. Pulmonary vessels ranging between 2 and 10 mm in diameter were extracted and labeled as either arteries or veins by an independently validated fully automated algorithm. Segmentation labels were validated by a radiologist. **Results**: The segmentation algorithm reached a median accuracy of 90%, aligning with the radiologist’s assessments. Vessel density of pulmonary arteries with diameters between 6 and 10 mm was higher in patients with versus those without PH (median [inter-quartile range]: 8.9 [6.1–10.8] 1/L vs. 6.2 [3.1–7.0] 1/L; *p* = 0.007). Artery-to-vein ratio was higher in PH (1.32 [0.93–2.06] vs. 0.88 [0.48–1.17], *p* = 0.004). The artery-to-vein ratio for vessels with diameters between 6 and 10 mm identified PH with an AUC of 0.73 (95% CI: 0.60–0.87). Combining this readout with the DMPA resulted in a numerically higher AUC (sole DMPA AUC: 0.79 (95% CI: 0.68–0.90)) vs. DMPA + artery-to-vein ratio for vessels with diameters of 6–10 mm: 0.81 (95% CI: 0.71–0.92); however, this improvement was not statistically significant (*p* = 0.4). **Conclusions**: PH is associated with an increased ratio of peripheral pulmonary arteries to veins within the 6–10 mm diameter range. Pulmonary vascular morphology may complement the established morphological criterion of MPA diameter and improve the diagnostic accuracy of PH on CT.

## 1. Introduction

Pulmonary hypertension (PH) is a serious and progressive disease that, with a global prevalence of about 1%, represents a significant global health challenge [1]. PH encompasses a group of diseases characterized by an elevated mean pulmonary arterial pressure (mPAP) at rest, as measured by right heart catheterization (RHC). Recently updated guidelines lowered the threshold for diagnosing PH from >25 mmHg to >20 mmHg [1,2,3,4]. PH is associated with poor outcomes, including a threefold increase in mortality compared to matched cohorts without PH, and a mean five-year survival of 72.5% [5]. This poor prognosis is partly due to the long delay between the onset of symptoms and establishing the diagnosis of PH. Thus, early diagnosis is crucial, as it enables treatment when it offers the greatest potential benefit [6].

The diagnostic approach and management of PH aim to achieve two key objectives: First, early identification of patients who may have PH, followed by prompt referral to an expert center for further evaluation and confirmation. Second, etiological clarification and subclassification of PH to aid in prognosis assessment and guide targeted therapy [1]. However, CT pulmonary angiography increasingly moves beyond main pulmonary artery diameter measurements toward comprehensive, quantitative assessment of the pulmonary vasculature and right heart to improve early diagnosis and risk stratification in pulmonary hypertension [7,8,9].

While RHC remains the gold standard for diagnosing PH, its invasive nature limits its practicality in cases where the indication is uncertain. Therefore, there is a clear need for non-invasive methods to aid in identifying patients who are likely to have PH and would benefit from undergoing RHC [10]. Besides transthoracic echocardiography, which plays a central role in determining the need for and timing of RHC in suspected PH cases [7], various imaging biomarkers have been introduced to aid in PH screening. These include an increased diameter of the main pulmonary artery (DMPA), an increased MPA-to-ascending aorta diameter ratio (DMPA/DAo), a segmental artery-to-bronchus ratio of ≥1:1 in three or four lobes, and enlargement of the right heart chambers [3,10,11,12,13,14,15,16]. Recent findings by Liu et al. demonstrated that, under the revised hemodynamic definition of PH, an increased DMPA (≥30 mm) has a sensitivity of 83.1% and a specificity of 90.4% for predicting PH [17]. In addition, modern imaging statements emphasize that advanced CT techniques, including quantitative vascular analysis, may complement echocardiography and invasive hemodynamics for screening and phenotyping of pulmonary hypertension [6].

Pathophysiologically, central vascular dilation results from increased transmural pressure in the pulmonary vasculature due to elevated pulmonary vascular resistance [18,19]. These changes can also lead to vascular pruning and alter the tortuosity of the peripheral pulmonary vessels [20,21]. Pulmonary vascular pruning, characterized by loss of small distal vessels and relative proximal dilation, is a key structural manifestation of pulmonary vascular remodeling and can be quantified on CT [20,22]. Studies have shown that these effects on peripheral lung vascular morphology can be objectively assessed on CT [20,21,23]. Specifically, automated artery–vein separation algorithms enable separate quantification of arterial and venous vascular trees, facilitating derivation of metrics such as the artery-to-vein ratio as potential imaging biomarkers [24]. Such automated pulmonary vessel analysis pipelines have been successfully applied across diseases including COPD and interstitial lung disease, supporting their methodological robustness and clinical relevance [25].

The aim of the current study was to assess whether the morphology of automatically segmented peripheral pulmonary arteries and veins can help in diagnosing PH based on the recently updated hemodynamic definition. Specifically, we assessed the diagnostic accuracy to diagnose PH using conventional vascular parameters (e.g., pulmonary artery diameter) and automated pulmonary vessel metrics in the largest cohort for this question to date.

## 2. Materials and Methods

### 2.1. Study Population

This study was approved by the University of Cologne institutional review board (22-1299-retro, 16 September 2022). The necessity for informed consent was waived due to the retrospective design of the study. All clinical investigations were conducted in accordance with the Declaration of Helsinki.

This single-center, retrospective study included 229 patients who underwent right heart catheterization (RHC) and additional testing due to suspected pulmonary hypertension (PH) between May 2016 and February 2022, including CTPA within 30 days of RHC. PH was defined as a mean pulmonary arterial pressure at rest > 20 mmHg, measured by RHC [1,4]. All patients underwent CT pulmonary angiography (CTPA) as part of their clinical evaluation. The final diagnosis was established by expert consensus, based on all available diagnostic data, including CT imaging. Inclusion criteria were: (1) right heart catheterization confirming or excluding PH; and (2) CTPA performed. Exclusion criteria were: (1) incomplete hemodynamic data; (2) poor CTPA image quality; (3) >30-day interval between RHC and CTPA; (4) failed vessel segmentation; and for analyses of arteries and veins, (5) an artery–vein segmentation accuracy <80%, as validated by an experienced radiologist.

### 2.2. Right Heart Catheterization, Echocardiography, Laboratory and Exercise Testing

Right heart catheterization was performed via internal jugular access using a Swan–Ganz catheter. Key hemodynamic variables measured included mPAP, pulmonary arterial wedge pressure (PAWP), mixed venous oxygen saturation (SvO_2_), mean right atrial pressure (RAP), cardiac index (CI), and pulmonary vascular resistance (PVR). Exercise capacity was quantified via the 6-min walk distance (6MWD), and plasma NT proBNP levels were also obtained.

Transthoracic echocardiography adhered to the American Society of Echocardiography/European Association of Cardiovascular Imaging guidelines for right heart assessment [26], including planimetric measurement of the right atrial (RA) area in the apical four chamber view at end systole. Tricuspid annular plane systolic excursion (TAPSE) and estimated systolic pulmonary artery pressure (sPAP) were used to calculate the TAPSE/sPAP ratio, reflecting right ventricular–pulmonary arterial coupling.

### 2.3. CTPA Image Acquisition and Reconstruction

CTPA data were acquired using a clinically available spectral detector CT scanner (IQon, Philips Healthcare, Best, The Netherlands) following the department’s standard protocol, in line with current recommendations [27]. All patients received an intravenous bolus of 50 mL contrast media (300 mg iodine/mL, Accupaque, GE Healthcare, Chicago, IL 60661, USA), followed by a 40 mL NaCl chaser, both administered at a flow rate of 4 mL/s. Scanning was initiated 4.9 s after automatic triggering detected an attenuation of 150 Hounsfield units (HU) in the main pulmonary artery (MPA). The acquisition parameters were slice collimation 64 × 0.625 mm; rotation time 0.33 s; tube potential 120 kV; tube current 75 mAsref, with automatic tube current modulation activated. A soft tissue reconstruction kernel (Spectral, B, Philips Healthcare) was used for all images, which were reconstructed in axial orientation every 0.5 mm, with a slice thickness of 1 mm and a matrix size of 512 × 512. The DICOM images were converted to MetaImages (.mha). No additional smoothing or denoising was applied beyond the spectral reconstruction kernel used during image acquisition.

### 2.4. Vessel Segmentation

**Figure 1:**
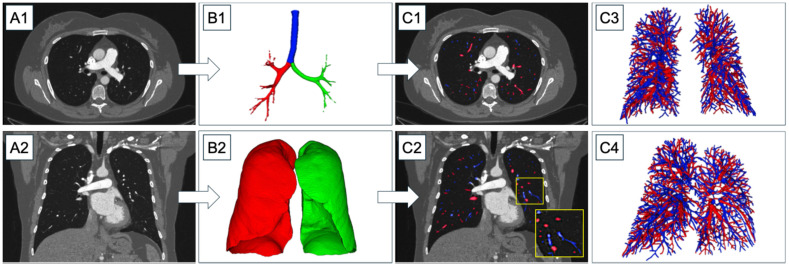
Flowchart of the fully automated vessel segmentation and artery/vein separation. (**A1**) and (**A2**): Sample CT image of a patient without PH in axial (**A1**) and coronal (**A2**) views. (**B1**): Corresponding airway segmentation, blue = trachea, red/green = right/left bronchi. (**B2**): Corresponding lung segmentation with corresponding colour scheme to (**B1**). (**C1**) and (**C2**): The results of the A/V segmentation displayed as overlays on the CT image in axial (**C1**) and coronal (**C2**) views. (**C3**) and (**C4**): 3D renderings of the pulmonary vessels. Arteries are depicted in blue and veins in red. PH, pulmonary hypertension.

Vessel segmentation and artery/vein separation were carried out using an in-house-developed rule-based algorithm with previous application in PH patients [28,29]. The fully automatic integer-programming-based method separated arteries and veins in thoracic computed tomography images by combining local as well as global properties of pulmonary vessels. In brief, lungs and airways were identified based on their attenuation. A multiscale vessel enhancement filter was applied to generate images with a high response for tubular structures within a set diameter range of 2 to 10 mm, along with an estimate of the tube orientation. Connecting regularly spaced maxima in these images, optimized vessel trees were constructed, preferentially selecting those connections that do not cross lung parenchyma and do not make sudden changes in direction or diameter. Arteries and veins were identified based on their approximate uniform and maximally intertwined distribution in the lungs and the characteristic parallel course of arteries alongside bronchi (Figure 1). Quantitative validation of the underlying algorithm has been reported previously, demonstrating a median voxel-based overlap of 96.3% with the manual reference segmentations and very few non-vascular structures (median value: 0.9%) and merged subtrees (median value: 0.6%). While the vessel segmentation algorithm was originally developed on images from a Siemens dual-energy CT scanner, it was later applied in several studies on images from a variety of CT scanners from other vendors with comparable segmentation accuracy [28,30,31,32,33]. More information on the algorithm can be found in Supplementary Figure S1.

A radiologist with 4 years of experience (RJG) validated all artery/vein segmentations. Scans with less than 80% correctly labeled vessels were excluded from vessel-type specific analyses. Visual inspection was chosen as the method for validation since it was found to be a good estimator for the manual labelling of individual vessel trees [31]. Further, this allowed general quality control to exclude insufficient segmentations.

The following parameters were assessed in this study: the number of vessel segments and vascular volume for the entire vasculature, including both arteries and veins, across the whole lung (right and left lung combined). Vessel density and normalized vessel volume were calculated by relating these values to the total lung volume. Vessel tortuosity was assessed using the sum-of-angles metric (SOAM) [34], calculated by summing up the angles between adjacent sections of a vessel segment and dividing the result by the length of the vessel segment, which is represented by the cumulative length of the sections used. The median tortuosity was used for analysis. To investigate whether smaller or larger vessels carried information related to an increased mPAP, the number of vessel segments was further analyzed by diameter ranges: 2 to 4 mm, 4 to 6 mm, and 6 to 10 mm. These ranges were selected to ensure sufficient vessel numbers within each category. Differences between arteries and veins, as well as ratios of arteries to veins, were calculated for all parameters. In addition, the diameters of the main pulmonary artery (DMPA) and the ratio of the MPA to the ascending aorta diameter (DMPA/DAo), known markers for the presence of PH [13,16,27], were measured from transverse CT images by a radiologist with 4 years of experience (RJG).

### 2.5. Statistical Analysis

Statistical analysis was performed in R (R Core Development Team, version 4.4.1), using RStudio (RStudio, Version 2023.6.1) [35]. Categorical data were analyzed with χ^2^ tests. The Wilcoxon rank sum test was used to determine normal distribution of continuous data. Since most data were not normally distributed, we applied nonparametric tests throughout this study. Hence, differences between groups were tested with Kruskal–Wallis rank sum tests and, if applicable, posthoc Dunn’s rank sum test with Bonferroni adjustment. Correlations of readouts with clinical parameters were determined with Spearman correlation coefficients. The abilities of the readouts to identify patients with PH were analyzed with ROC analyses using the pROC package. To test whether combinations of readouts can improve diagnostic accuracy, the methodology proposed by Pepe et al. was applied [36]. Differences in AUCs were assessed using the DeLong test [37], and *p*-values < 0.05 were considered significant. Data generated or analyzed during the study are available from the corresponding author by request.

## 3. Results

### 3.1. Patient Characteristics

**Figure 2:**
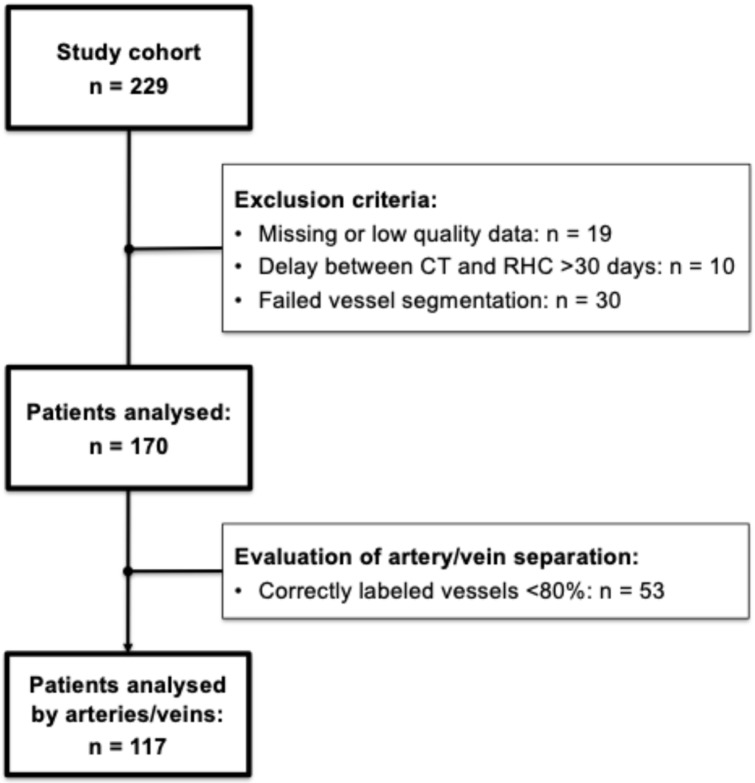
Stepwise overview from the original cohort to the final study cohort and the A/V subcohort, including exclusion criteria and patient numbers. A/V, arteries/veins. RHC, right heart catheterization.

Out of 229 patients, 19 were excluded due to low image quality or missing data. Ten patients were excluded due to >30 days delay of CT and RHC. Vessel segmentation failed in another 30 patients, mainly due to issues with airway segmentation. Therefore, the final cohort consisted of 149 patients with PH and 21 patients without PH. The A/V separation algorithm correctly labeled arteries and veins with a median accuracy of 90% (interquartile range: 75–98%). Mislabeling exceeding 20% led to the exclusion of 53 patients (31.1%) from the vessel-type-specific analysis, resulting in the A/V subcohort (Figure 2). There were no differences between the patients with and without PH regarding age (median [inter-quartile range]: PH, 69 [57–77] years vs. no PH, 59 [52–69] years, *p* = 0.06) or sex (PH: 88/61 f/m vs. no PH: 12/9 f/m, *p* = 1.0). Table 1 summarizes the patient characteristics. Importantly, there were no differences between included and excluded patients, i.e., original cohort vs. final cohort regarding clinical parameters (Supplementary Table S1).

### 3.2. Lung Vessel Segmentation

Overall, the algorithm identified a median 1843 [interquartile range: 1540–2151] vessel segments in all CT images. This corresponds to a vessel density of 442 [364–485] vessel segments per liter of lung volume (1/L). We found several moderate to strong correlations between lung vascular morphology readouts and pulmonary hemodynamics (Table 2). The strongest correlation was observed between the ratio of arteries to veins, with a diameter of 6–10 mm and the cardiac index (CI) (r = −0.59, *p* < 0.001). The vessel density of arteries in the same diameter range showed the strongest association with mPAP among all automatic readouts (r = 0.42, *p* < 0.001). Additionally, both the DMPA and the DMPA/DAo ratio showed strong correlations with the mPAP (DMPA: r = 0.51, *p* < 0.001; DMPA/DAo: r = 0.52, *p* < 0.001).

### 3.3. Differences Between PH and Non-PH Patients in Pulmonary Vessel Morphology

**Figure 3:**
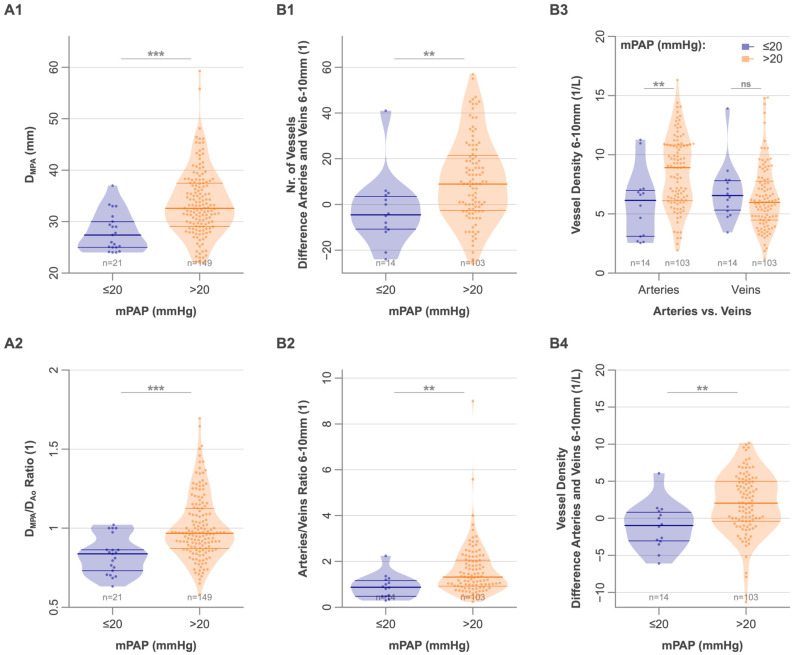
Violin plots of pulmonary vascular morphology in patients with mPAP ≤20 mmHg and >20 mmHg (PH). (**A1**): DMPA shows larger diameters with higher mPAP. (**A2**): Ratio of DMPA to DAo indicates relative DMPA enlargement in PH patients. (**B1**): Difference in the number of arteries and veins (6–10 mm) shows a shift toward more arteries at higher mPAP. (**B2**): Artery-to-vein ratio (6–10 mm) with higher values among patients with PH. (**B3**): Vessel density (1/L) for arteries and veins (6–10 mm) demonstrates increased arterial but unchanged venous density among patients with PH. (**B4**): Difference in vessel density between arteries and veins (6–10 mm) highlights arterial predominance among patients with PH. mPAP, mean pulmonary artery pressure; DMPA, diameter main pulmonary artery; DAo ascending aorta diameter; PH, pulmonary hypertension. **, *** indicate *p* < 0.01, and *p* < 0.001.

**Figure 4:**
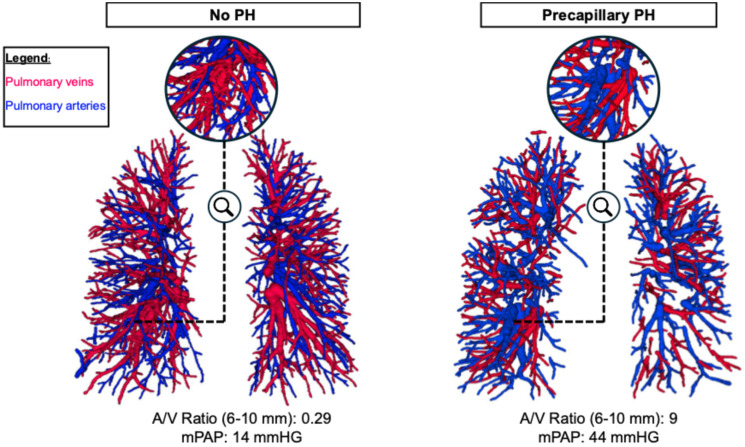
3D volume rendering of artery and vein segmentation (arteries: blue, veins: red) in a patient without PH (mPAP, 14 mmHg) and in a patient with precapillary PH (mPAP, 44 mmHg). Zoomed-in sections highlight peripheral vascular differences, illustrating the higher A/V ratio in PH (0.29 vs. 9, respectively). The two patients represent extreme examples. Note that this figure is only for illustrative purposes; the presented algorithm is not intended for visual PH diagnostic. A/V Ratio, artery-to-vein ratio. mPAP, mean pulmonary artery pressure; PH, pulmonary hypertension.

While there was no difference between patients with and without PH in the overall number of vessel segments or the vessel density (Table 3), patients with PH exhibited an increased number (36 [27–54] vs. 27 [11–34], *p* = 0.009) and density of pulmonary arteries measuring 6–10 mm diameter (8.9 [6.1–10.8] 1/L vs. 6.2 [3.1–7.0] 1/L, *p* = 0.007). As the vessel density for veins in this diameter range was similar for the two groups, the difference in vessel density between arteries and veins in this diameter range was higher in PH patients (2.0 [−0.4–5.0] 1/L vs. −1.0 [−3.1–0.8] 1/L, *p* = 0.004), leading to a higher artery-to-vein ratio in these vessels (1.32 [0.93–2.06] vs. 0.88 [0.48–1.17], *p* = 0.004) (Figure 3 and Figure 4). Both the DMPA and the DMPA/DAo ratio were higher in patients with PH (DMPA: 32.7 [29.4–37.5] mm vs. 27.4 [25.0–30.0] mm, *p* < 0.001; DMPA/DAo: 0.97 [0.87–1.13] vs. 0.84 [0.73–0.86], *p* < 0.001). Interestingly, in this cohort, the vessel tortuosity was higher in patients without PH than in patients with PH (SOAM: 0.14 [0.13–0.15] rad/mm) and veins (0.13 [0.12–0.14] rad/mm) compared to non-PH patients (arteries: 0.16 [0.15–0.16], veins: 0.15 [0.14–0.16]; *p* < 0.001 for both, Table 3).

**Figure 5:**
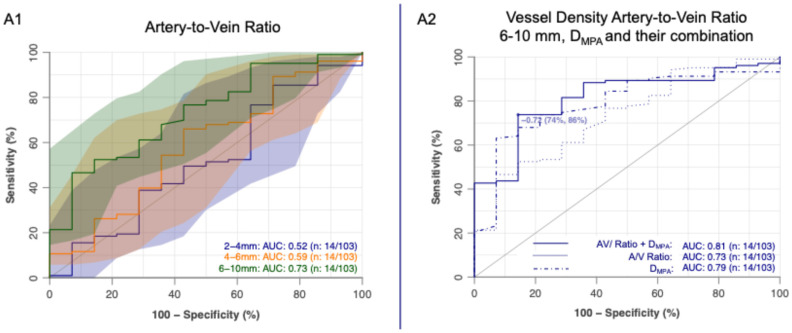
(**A1**) ROC analysis of the artery-to-vein (A/V) ratio across different peripheral vessel diameter ranges (2–4 mm, 4–6 mm, and 6–10 mm). (**A2**) ROC analysis of the A/V ratio in the 6–10 mm diameter range, the main pulmonary artery diameter (D_MPA), and their combined diagnostic performance. ROC, receiver operating characteristic; A/V ratio, artery-to-vein ratio; D_MPA, diameter of the main pulmonary artery.

The artery-to-vein ratio for vessels with diameters of 6–10 mm identified PH patients with an AUC of 0.73 (95% CI: 0.60–0.87) (Figure 5A1). Combining this metric with the DMPA or the DMPA/DAo ratio resulted in numerically higher AUCs compared to either measure alone. Specifically, the AUC increased from 0.79 (95% CI: 0.68–0.90) for DMPA alone to 0.81 (95% CI: 0.71–0.92) when combined with the artery-to-vein ratio (Figure 5A2). Similarly, the AUC for DMPA/DAo alone (0.73 [95% CI: 0.59–0.88]) increased to 0.83 (95% CI: 0.73–0.92) with the addition of the artery-to-vein ratio, though without reaching statistical significance (*p* = 0.20).

## 4. Discussion

In this retrospective study, we assessed the diagnostic value of automated, quantitative analysis of peripheral pulmonary vascular morphology on CTPA in patients with and without PH, applying the revised hemodynamic definition of the recently updated guidelines to PH diagnosis [1]. The artery-to-vein ratio of pulmonary arteries in the 6–10 mm diameter range showed moderate to strong correlations with hemodynamic parameters, particularly mPAP and CI, and emerged as the strongest marker for identifying PH. Combining them with established parameters such as DMPA and the DMPA/DAo ratio resulted in a numerical increase in AUC, indicating potential added diagnostic value.

CT imaging provides important insights into PH [1,9,38,39,40], inherently capturing the heart, pulmonary vascular tree, and lung parenchyma. Enlargement of the MPA and an increased DMPA/DAo ratio are the best-established CT signs of PH [1,27], are easy to measure, and retain value under the revised definition lowering the mPAP threshold from >25 to >20 mmHg [1,2,4,17]. Large registry-based CT analyses have shown that specific CT patterns in pulmonary arterial hypertension correlate with clinical phenotype and outcome, underlining the value of detailed morphologic assessment [41]. In our cohort, DMPA and DMPA/DAo ratio were increased in PH and correlated most strongly with mPAP, in line with prior work [17]. However, their diagnostic accuracy was lower than reported by Liu et al., reflecting the variability seen in the literature [27,42,43,44,45,46,47,48,49], likely due to differences in patient selection, pre-test probability, diagnostic criteria, and measurement techniques [27,50,51]. Consequently, the current Fleischner Society position paper on imaging of PH in adults considers both the MPA diameter and DMPA/DAo ratio insufficient as stand-alone criteria for PH screening [27].

These drawbacks may also reflect a more fundamental limitation: such central artery measurements overlook the peripheral pulmonary vasculature—the primary site of the pathophysiological changes in PH [52]. This recognition has prompted efforts to improve diagnostic accuracy by extending quantitative analysis beyond the central pulmonary arteries [5,6,20,21,23]. Advanced tools capable of automated artery–vein separation can quantify vessel diameters, density, and tortuosity, offering access to this information.

Using an in-house algorithm, we found PH patients exhibited a higher artery-to-vein ratio and greater arterial density in 6–10 mm vessels, consistent with Rahaghi et al. [6,20,23]. This shift likely reflects distal vascular pruning, a hallmark of PAH and other PH-associated conditions [1,53]. Importantly, we do not assume dynamic appearance or disappearance of vessels in the 2–10 mm range but rather capture altered segments of proximal (thinning) or distal vessels (dilation).

To our knowledge, this is the largest cohort to date evaluating the value of segmentation of the peripheral pulmonary vascular tree in addition to conventional CT metrics considering the recently updated guidelines. Our approach integrates size-specific A/V morphology with established CT markers (DMPA, DMPA/DAo) to evaluate their combined diagnostic performance. Another key innovation of this study is the targeted assessment of artery–vein ratios within the 6–10 mm vessel range, which represents proximal segments most susceptible to PH-related remodeling.

We found that combining DMPA or the DMPA/DAo ratio with the artery-to-vein ratio in 6–10 mm vessels modestly increased AUCs, suggesting added diagnostic value. The non-significant increase in AUC likely reflects limited statistical power due to the reduced A/V cohort after exclusion of scans with insufficient segmentation quality. Nevertheless, the numerical improvement suggests complementary diagnostic information beyond central vessel measurements, which may be clinically relevant, particularly in borderline cases or early disease stages. However, routine use is limited by the need for algorithm-driven processing, technical validation, and manual oversight. Exclusion of 31.1% of patients underscores these challenges. The main causes of mislabeling were: (1) swapping artery/vein labels in whole lung segments or lobes, often influenced by correct labelling in an adjacent lobe without direct vascular connection; (2) incomplete airway segmentation in patients with severe parenchymal disease; and (3) markedly heterogeneous contrast enhancement. In contrast, MPA diameter measurement remains simple, shows low inter-reader variability even among non-experts [54], and is widely applicable. On the other hand, the artery-to-vein ratio in the 6–10 mm diameter range was recently reported to correlate significantly with prognostic markers in PH and to differentiate between low- and high-risk mortality groups [28]. As such, it may also prove valuable in downstream clinical decision-making and patient management. This is in line with prior work, pointing out that quantitative vascular indices may improve prognosis in patients with pulmonary hypertension when combined with conventional CT markers such as main pulmonary artery diameter and right heart size [55].

In contrast to previous studies that consistently reported increased tortuosity of the pulmonary arteries in PH [20,23], our cohort demonstrated lower arterial tortuosity in PH patients compared to controls. In line with this, vascular pruning has also been described in smokers and patients with COPD, where reduced small-vessel blood volume correlates with airflow limitation [22]. This apparent discrepancy may be attributed to differences in control group composition. In our study, the control group consisted of patients who underwent RHC due to clinical suspicion of PH, with a median mPAP of 17 mmHg, but in whom PH was ultimately ruled out. Unlike healthy volunteers used in some prior studies [23], our controls were affected by various pulmonary comorbidities that may independently influence vascular morphology. Specifically, a subset had interstitial lung disease related to systemic sclerosis or chronic obstructive pulmonary disease, both of which are associated with pulmonary vascular remodeling [33,56] and an elevated risk for PH. Additionally, several controls exhibited chronic thromboembolic changes in the pulmonary arteries without meeting the hemodynamic criteria for PH. These comorbidities may contribute to an increased tortuosity despite the absence of overt pulmonary hypertension. Taken together, these findings indicate that pulmonary artery tortuosity may not be a specific marker of PH but rather a more general indicator of pulmonary vascular pathology.

In addition to the retrospective design, our cohort, similar to previous studies [17,50,57], exclusively included patients with a high clinical suspicion of PH, both in the PH and control groups. While this selection reflects the clinical context in which RHC and CTPA are typically performed, it likely increased the overall prevalence of pulmonary vascular abnormalities. Consequently, the diagnostic performance metrics observed here are unlikely to directly translate to lower-prevalence settings or populations with milder disease, and should be validated in broader, unselected cohorts.

Several other factors must be considered. The segmentation algorithm was developed in-house and is not commercially available, which may limit its generalizability and broader clinical application. Furthermore, the automated vessel analysis excluded the central pulmonary vasculature and was restricted to vessels larger than 2 mm in diameter, potentially affecting the overall diagnostic performance. Additionally, as our study focused specifically on pulmonary vascular morphology, other CT-based indicators of PH—such as signs of right heart pressure overload—were not included in the analysis. Finally, since our cohort does not reflect the true prevalence of PH in the general population, conclusions regarding the diagnostic value and predictive performance of the proposed morphological markers are inherently limited and require prospective validation in more representative cohorts.

Several deep-learning-based methods for automated pulmonary artery–vein separation have been proposed recently. For example, HiPaS uses super-resolution and cascaded segmentation networks pretrained on large CT datasets, achieving Dice similarity coefficients of 89–92% for arteries and veins in non-contrast CT [58]. VLSOM [59] employs a 3D U-Net with centerline extraction and weighted losses, reporting centerline Dice scores of 0.89–0.93 on contrast-enhanced and non-contrast CT from multiple vendors but lacks in-depth clinical validation. Earlier approaches, such as 3D CNNs with graph-cut optimization reached voxel accuracies around 94% but required more manual tuning, complicating generalized clinical application [60].

In comparison, the knowledge-based integer programming algorithm applied here relies on anatomical rules like uniform artery–vein distribution and bronchial proximity of arteries, yielding a median voxel overlap of 96.3% with manual reference on 25 thoracic CTs and consistent performance across vendors without training data. While deep learning methods excel in scalability to low-contrast images and large datasets, they demand extensive annotations and may overfit to specific scanners. The rule-based approach offers greater interpretability and lower computational needs, making it suitable for clinical cohorts with variable image quality, and hence, facilitate clinical application.

In conclusion, the artery-to-vein ratio in vessels measuring 6–10 mm provides incremental diagnostic value beyond MPA diameter for predicting PH when applying the revised hemodynamic definition. Given its prognostic relevance, this parameter may represent a valuable imaging biomarker in PH. In contrast, vascular tortuosity appears more susceptible to the influence of pulmonary comorbidities. While this limits its utility as a specific screening parameter for PH, it may serve as a more sensitive marker for detecting pulmonary vascular alterations beyond PH alone.

## Figures and Tables

**Table 1:** Patient characteristics.

Parameter	no PH	PH	*p*
Sex (F/M)	12/9	88/61	*p* = 1.0
Age (yrs; n = 170; n = 21/149)	59 [52–69]	69 [57–77]	*p* = 0.06
BSA (m^2^; n = 127; n = 13/114)	2.12 [2.00–2.48]	1.87 [1.69–2.06]	*p* = 0.02
mPAP (mmHg; n = 170; n = 21/149)	17 [16–19]	38 [29–48]	*p* < 0.001
PVR (WU; n = 158; n = 16/142)	1.4 [1.0–2.4]	5.5 [3.3–8.2]	*p* < 0.001
PAWP (mmHg; n = 163; n = 21/142)	9 [8–10]	14 [10–18]	*p* < 0.001
6MWD (m; n = 123; n = 16/107)	366 [309–489]	375 [271–444]	*p* = 0.4
NT-pro BNP (ng/L; n = 160; n = 20/140)	131 [83–240]	665 [241–1915]	*p* < 0.001
CI (L/min/m^2^; n = 136; n = 15/121)	2.34 [2.08–2.71]	2.30 [1.90–2.77]	*p* = 0.7
SvO_2_ (%; n = 159; n = 20/139)	69 [64–73]	66 [60–70]	*p* = 0.06
RAP (mmHg; n = 158; n = 19/139)	5 [2–7]	8 [5–11]	*p* < 0.001
RA area (cm^2^; n = 157; n = 19/138)	17 [14–22]	22 [19–27]	*p* = 0.002
FAC (%; n = 39; n = 4/35)	39 [35–40]	29 [21–34]	*p* = 0.14
TAPSE (mm; n = 160; n = 19/141)	22 [20–27]	20 [17–24]	*p* = 0.07
TAPSE/sPAP (mm/mmHg; n = 136; n = 16/120)	0.6 [0.4–0.8]	0.3 [0.2–0.5]	*p* < 0.001
V_Lung_ (L; n = 21/149)	4.2 [3.6–5.6]	4.4 [3.4–5.3]	*p* = 0.9

Data presented as either counts or median [interquartile range]. 6MWD. 6-min walking distance; BSA. body surface area; CI. cardiac index; FAC. Fractional Area Change; mPAP. mean pulmonary artery pressure; n. number of subjects; NT-proBNP. plasma levels of N-terminal pro-brain natriuretic peptide; PAWP. pulmonary artery wedge pressure; PH. pulmonary hypertension; PVR. pulmonary vascular resistance; RA. right atrium; RAP. right atrial pressure; sPAP. systolic pulmonary artery pressure determined with echocardiography; SvO_2_. mixed venous oxygen saturation; TAPSE. tricuspid annular plane systolic excursion; V_Lung_. lung volume from computed tomography images; WU. Wood-Units.

**Table 2:** Correlations of lung vascular morphology readouts and hemodynamics.

	Nr. of Vessels				Vessel Volume	SOAM [rad/mm]
	All Diameters	2–4 mm	4–6 mm	6–10 mm	All Diameters	All Diameters
All vessels:						
Age (yrs)						
BSA (m^2^)	0.23 ** (127)	0.19 * (127)	0.37 *** (127)	0.35 *** (127)	0.47 *** (127)	0.39 *** (127)
mPAP (mmHg)		−0.13 (170)				−0.28 *** (170)
PVR (WU)	−0.16 (158)	−0.15 (158)			−0.21 ** (158)	−0.29 *** (158)
PAWP (mmHg)		−0.15 (163)				
CI (L/min/m^2^)						
Arteries:						
Age (yrs)		−0.16 (117)		0.23 * (117)		
BSA (m^2^)			0.32 ** (89)	0.32 ** (89)	0.36 *** (89)	0.35 *** (89)
mPAP (mmHg)			0.15 (117)	0.25 ** (117)		−0.30 ** (117)
PVR (WU)				0.21 * (109)		−0.27 ** (109)
PAWP (mmHg)	−0.18 (113)	−0.21 * (113)				
CI (L/min/m^2^)				−0.24 * (95)		
Veins:						
Age (yrs)				−0.23 * (117)		
BSA (m^2^)			0.24 * (89)	0.21 (89)	0.38 *** (89)	0.30 ** (89)
mPAP (mmHg)				−0.24 ** (117)	−0.17 (117)	−0.33 *** (117)
PVR (WU)	−0.24 * (109)	−0.22 * (109)	−0.28 ** (109)	−0.30 ** (109)	−0.33 *** (109)	−0.28 ** (109)
PAWP (mmHg)						
CI (L/min/m^2^)	0.29 ** (95)	0.24 * (95)	0.34 *** (95)	0.46 *** (95)	0.27 ** (95)	
Ratio arteries/veins:						
Age (yrs)		−0.21 * (117)	0.27 ** (117)	0.37 *** (117)	0.25 ** (117)	
BSA (m^2^)						
mPAP (mmHg)			0.26 ** (117)	0.41 *** (117)	0.30 *** (117)	
PVR (WU)	0.21 * (109)		0.40 *** (109)	0.45 *** (109)	0.44 *** (109)	
PAWP (mmHg)	−0.17 (113)	−0.20 * (113)				
CI (L/min/m^2^)	−0.26 * (95)		−0.46 *** (95)	−0.59 *** (95)	−0.47 *** (95)	

Data presented as Spearman correlation coefficient (*p*-value. number of patients). *, **, *** indicate *p*  <  0.05, *p*  <  0.01, and *p*  <  0.001, respectively. Ellipses indicate correlations with a *p*-value ≥ 0.1. BSA. body surface area; CI. cardiac index; mPAP. mean pulmonary artery pressure; n. number of subjects; PAWP. pulmonary artery wedge pressure; PVR. pulmonary vascular resistance; SOAM (rad/mm). sum of angles metric (radiant/millimeter); WU. Wood-Units.

**Table 3:** Differences in lung vascular morphology between patients with PH and patients without PH.

	no PH	PH	*p*
All vessels. all diameters			
Nr. of Vessels (1; n = 21/149)	1862 [1543–2068]	1840 [1539–2198]	*p* = 0.9
Vessel Density (1/L; n = 21/149)	443 [378–499]	442 [364–482]	*p* = 0.7
SOAM (rad/mm; n = 21/149)	0.15 [0.14–0.16]	0.14 [0.13–0.14]	*p* < 0.001
Arteries. all diameters			
Nr. of Vessels (1; n = 14/103)	883 [779–992]	942 [815–1226]	*p* = 0.3
Vessel Density (1/L; n = 14/103)	221 [197–250]	217 [187–259]	*p* = 0.9
SOAM (rad/mm; n = 14/103)	0.16 [0.15–0.16]	0.14 [0.13–0.15]	*p* < 0.001
Veins. all diameters			
Nr. of Vessels (1; n = 14/103)	839 [776–1001]	888 [759–1027]	*p* = 0.6
Vessel Density (1/L; n = 14/103)	214 [186–243]	207 [167–234]	*p* = 0.6
SOAM (rad/mm; n = 14/103)	0.15 [0.14–0.16]	0.13 [0.12–0.14]	*p* < 0.001
Arteries:			
Vessel Density—2–4 mm (1/L; n = 14/103)	195 [175–218]	190 [160–229]	*p* = 0.6
Vessel Density—4–6 mm (1/L; n = 14/103)	17 [14–20]	21 [17–24]	*p* = 0.08
Vessel Density—6–10 mm (1/L; n = 14/103)	6.2 [3.1–7.0]	8.9 [6.1–10.8]	*p* = 0.007
Veins:			
Vessel Density—2–4 mm (1/L; n = 14/103)	184 [165–212]	184 [143–208]	*p* = 0.6
Vessel Density—4–6 mm (1/L; n = 14/103)	21 [16–23]	19 [15–24]	*p* = 0.9
Vessel Density—6–10 mm (1/L; n = 14/103)	6.6 [5.3–7.8]	6.0 [4.5–7.8]	*p* = 0.4
Ratio arteries/veins:			
2–4 mm (1; n = 14/103)	1.09 [0.93–1.27]	1.09 [0.98–1.25]	*p* = 0.8
4–6 mm (1; n = 14/103)	0.93 [0.74–1.23]	1.05 [0.86–1.33]	*p* = 0.3
6–10 mm (1; n = 14/103)	0.88 [0.48–1.17]	1.32 [0.93–2.06]	*p* = 0.005
D_MPA_ (mm; n = 21/148)	27.4 [25.0–30.0]	32.7 [29.4–37.5]	*p* < 0.001
D_MPA_/D_Ao_ (1; n = 21/148)	0.84 [0.73–0.86]	0.97 [0.87–1.13]	*p* < 0.001

Data presented as median [interquartile range]. DMPA. diameter of the main pulmonary artery; DMPA/DAo. diameter ratio of main pulmonary artery over ascending aorta; SOAM (rad/mm). sum of angles metric (radiant/millimeter); PH. pulmonary hypertension.

## Data Availability

The dataset is available from the corresponding author upon reasonable request.

## Supplementary Materials

The following supporting information can be downloaded at: https://www.mdpi.com/article/10.3390/diagnostics16040619/s1, Table S1: Patient characteristics of included and excluded patients. Figure S1: Algorithm overview.

## Abbreviations

PH—Pulmonary Hypertension; mPAP—Mean Pulmonary Arterial Pressure; RHC—Right Heart Catheterization; CTPA—Computed Tomography Pulmonary Angiography; DMPA—Diameter of the Main Pulmonary Artery; DAo—Diameter of the Ascending Aorta; A/V ratio—Artery-to-Vein Ratio; PAWP—Pulmonary Arterial Wedge Pressure; SvO_2_—Mixed Venous Oxygen Saturation; RAP—Right Atrial Pressure; CI—Cardiac Index; PVR—Pulmonary Vascular Resistance; 6MWD—6-Minute Walk Distance; NT-proBNP—N-terminal pro–B-type Natriuretic Peptide; TAPSE—Tricuspid Annular Plane Systolic Excursion; sPAP—Systolic Pulmonary Artery Pressure; TAPSE/sPAP ratio—Ratio of TAPSE to sPAP; SOAM—Sum-of-Angles Metric; ROC—Receiver Operating Characteristic; AUC—Area Under the Curve.
