# Supplementary material for: Pulmonary Artery and Vein Morphology as an Imaging Biomarker for the Diagnosis of Pulmonary Hypertension
Source: Diagnostics (Basel). 2026 Feb 20;16(4):619. doi: 10.3390/diagnostics16040619 (PMC12939112; doi:10.3390/diagnostics16040619)
Supplement: Supplementary file 1 [file diagnostics-16-00619-s001.zip › Supplementary tables.pdf]

Supplementary Table S1: Patient characteristics of included and excluded patients

Parameter	Included	Excluded	*p*
Sex (F/M)	100/70	37/22	0.7
PH group (no PH/1/2/3/4/5)	35/29/43/19/43/1	16/11/6/6/13/1	0.3
Age (yrs; n=229; n=170/59)	68 [56-77]	68 [58-74]	0.3
BSA (m2; n=171; n=127/44)	1.88 [1,70-2,12]	1.97 [1.75-2.11]	0.3
mPAP (mmHg; n=226; n=170/56)	36 [26-46]	37 [25-49]	0.7
PVR (WU; n=211; n=158/53)	4.9 [2,8-7,8]	5.4 [2.7-9.1]	0.8
PAWP (mmHg; n=216; n=163/53)	13 [9-17]	12 [8-15]	0.2
NT-pro BNP (ng/L; n=216; n=160/56)	461 [174-1683]	602 [187-1901]	0.5
CI (L/min/m2; n=182; n=136/46)	2.31 [1,92-2,77]	2.44 [2.02-2.70]	0.7
SvO2 (%; n=208; n=159/49)	66 [61-70]	68 [62-71]	0.5
RAP (mmHg; n=208; n=158/50)	8 [5-10]	7 [4-11]	0.8
RA area (cm2; n=211; n=157/54)	22 [18-27]	21 [17-27]	0.6
FAC (%; n=48; n=39/9)	29 [21-36]	30 [26-31]	0.8
TAPSE (mm; n=214; n=160/54)	20 [17-24]	20 [18-23]	0.4
TAPSE/sPAP (mm/mmHg; n=169; n=136/33)	0.3 [0.2-0.5]	0.3 [0.2-0.4]	0.05

Data presented as either counts or median [interquartile range].

BSA. body surface area; CI. cardiac index; mPAP. mean pulmonary artery pressure; n. number of subjects; NT-proBNP. plasma levels of N-terminal pro-brain natriuretic peptide; PAWP. pulmonary artery wedge pressure; PH. pulmonary hypertension; PVR. pulmonary vascular resistance; RA. right atrium; RAP. right atrial pressure; sPAP. systolic pulmonary artery pressure determined with echocardiography; SvO_2. mixed venous oxygen saturation; TAPSE. tricuspid annular plane systolic excursion.
