# Supplementary figures and images for: Pulmonary Artery and Vein Morphology as an Imaging Biomarker for the Diagnosis of Pulmonary Hypertension
Source: Diagnostics (Basel). 2026 Feb 20;16(4):619. doi: 10.3390/diagnostics16040619 (PMC12939112; doi:10.3390/diagnostics16040619)

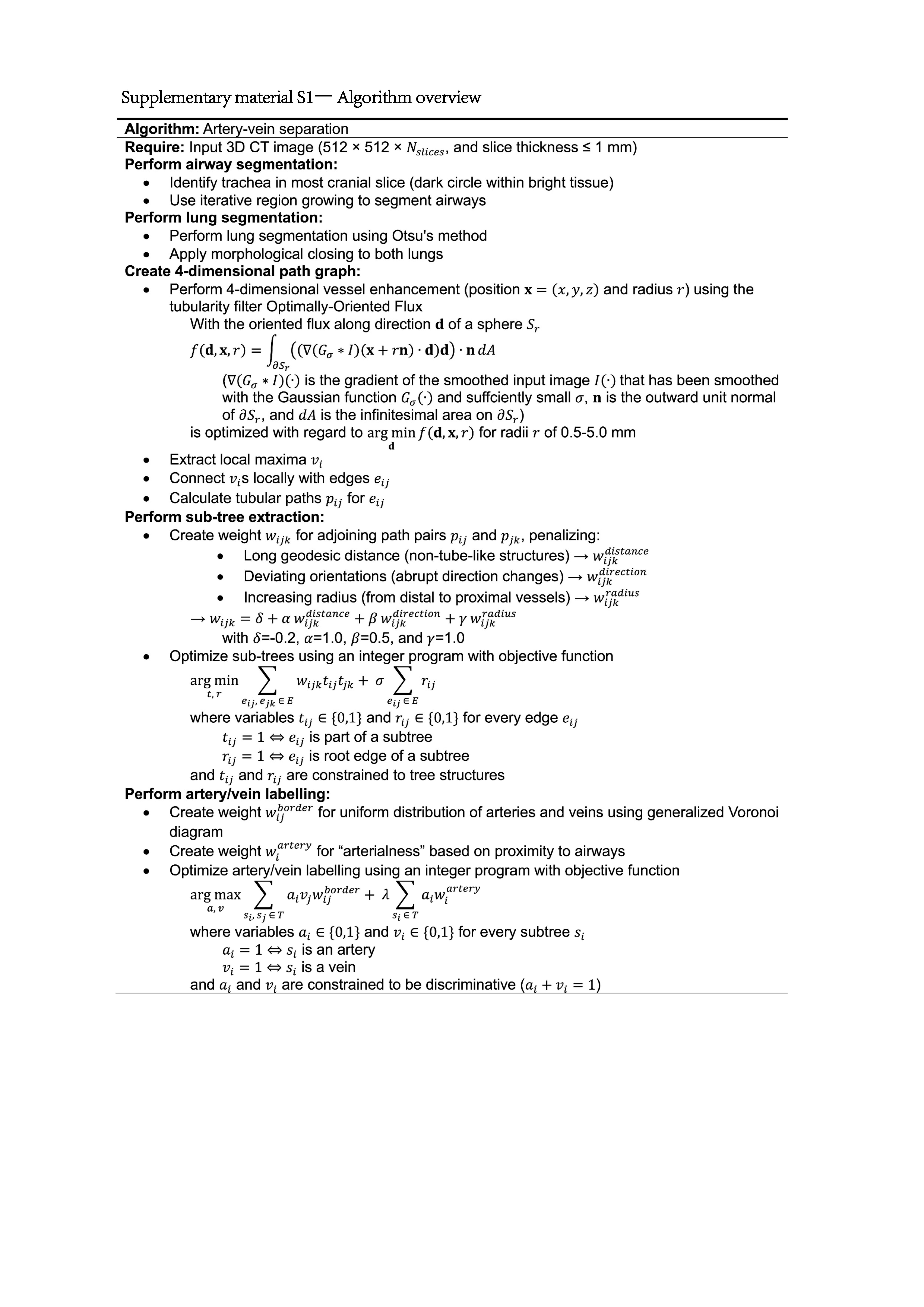

Supplement: Supplementary file 1 [file diagnostics-16-00619-s001.zip › Supplementary Figure S1.jpg]
